# Signs of Inequality? Variations in Providing Home Health Care Across Care Organizations and Across European Countries in the IBenC Study

**DOI:** 10.1177/1178632919837632

**Published:** 2019-04-18

**Authors:** Hein PJ van Hout, Lisanne van Lier, Stasja Draisma, Jan Smit, Harriet Finne-Soveri, Vjenka Garms-Homolová, Judith E Bosmans, Anja Declercq, Pálmi Jónsson, Graziano Onder, Henriëtte G van der Roest

**Affiliations:** 1 Department of General Practice and Elderly Care Medicine, Amsterdam UMC, Vrije Universiteit, Amsterdam Public Health, Amsterdam, The Netherlands; 2 GGZ InGeest Mental Health Organization, Amsterdam, The Netherlands; 3 National Institute for Health and Welfare, Helsinki, Finland; 4 Department of Economics and Law, HTW Berlin University of Applied Sciences, Berlin, Germany; 5 Department of Health Sciences, Faculty Science, Vrije Universiteit Amsterdam, Amsterdam Public Health, Amsterdam, The Netherlands; 6 Centre for Sociological Research, LUCAS—KU Leuven, Leuven, Belgium; 7 Department of Geriatrics, Landspitali University Hospital, University of Iceland, Reykjavík, Iceland; 8 Department of Gerontology, Neuroscience and Orthopedics, Università Cattolica del Sacro Cuore, Rome, Italy

**Keywords:** Equality, home health care, resource utilization, benchmarking, formal care, expected care

## Abstract

Most countries aim to allocate home health care to those in need in a fair and equal way. Equal allocation implies that the amount of home care a person receives would reflect the level of health impairment and the need for resources. It is not clear whether countries succeed in attaining this. Our objective was to explore signs of (un)equal home health care provisioning across care organizations and across European health countries. We used data of the IBenC study collected from 2718 older community care recipients from 33 organizations in 6 Western European countries (www.ibenc.eu). We benchmarked differences of provided and expected formal care time across organizations and countries. Expected formal care hours were estimated by multiplying the overall sample’s mean formal hours with recipients’ case mix weights from interRAI’s resources utilization group profiles. We found substantial variations in provided formal care time among organizations both within and across countries that could not be explained by the case mix differences of recipients. This implied presence of inequality of home care provisioning. These findings may alert professionals and policy makers striving for equal home health care provisioning for dependent older persons.

## Introduction

Most countries aim to allocate home health care to those in need in a fair and equal way. The amount of care provided to a person would then reflect the level or severity of health impairment. It is not clear whether health authorities and care organizations succeed in attaining this. Allocation criteria for community care may vary not only between countries but also between municipalities. Also, criteria may allow for more or less strict interpretation.^1^ It is also not clear in what way the availability of informal care plays a role in care allocations.

About one-fifth of persons 50 years and older who live independently receive some form of care, and about one-fifth of the total amount of care concerns formal care.^2,3^ Home health care constitutes the cornerstone of formal community care for dependent older persons in most European countries. Home health care is provided by health professionals and usually comprises home health support and nursing care.^1,4^

Most home health care recipients receive informal care.^2,4,5^ Informal care can be defined as unpaid help provided by members of a person’s social network.^6^ Informal care is often provided by close relatives and may comprise instrumental activities of daily living such as help with preparing meals, shopping, household activities, and supervision.^7,8^ Many informal caregivers are willing to offer assistance for such daily care needs. However, when care needs comprise basic self-care (care for bathing, eating, toileting), complex issues (eg, wound care and medication intake), or become more intensive (eg, more than 11 h a week), most informal carers are unable or unwilling to provide this care.^9^ Despite this, many informal caregivers continue at the cost of their own health and many experience severe burden, or even develop a depressive or anxiety disorder over time.^10-12^

Earlier studies reported substantial variations in dependency levels of home care recipients across countries.^13^ In countries with relatively few long-term care facilities, persons are being cared for longer in the community until more advanced stages of dependency. This goes hand in hand with a higher proportion of informal care. Countries may also apply different criteria for access to and number of hours of home health care.^1,4^ Such criteria typically assess a person’s capacity for self-care, and decision making, but sometimes also consider availability of informal care.^1,4^

The availability of care staff varies across countries.^1^ For the 6 countries studied in the IBenC study, variations in available staff ranged from as much as 15.4 nurses per 1000 inhabitants in Belgium down to 6.3 nurses per 1000 inhabitants in Italy.^1^ In addition, the age dependency ratio, the ratio of the number of persons above 65 years against the number of persons in the usual working ages (from 15 to 64), was 26.7 in Europe, but varied from as low as 18.9 in Iceland up to 31.6 in Italy.^1,14^ This indicates that in Italy, fewer working adults care are available to care for a larger portion of dependent older persons.

Despite these well-documented variations, it is unclear whether and to what extent home health care provided to individual care recipients varies across and within countries and whether care allocation occurs in an equal and fair way.

To evaluate equality and fairness of the allocation of home health care, recipients’ clinical and needs profile should be taken into account as well as the availability of informal care. Persons with more advanced dependency can be expected to receive more care compared with persons with mild dependencies. Variations *across* countries can be expected if we consider differences in allocation of home care and availability of formal care resources. Variations *within* countries might be less desirable, provided policy making strives for fair allocation of home health care.

Many European countries adopted the “aging in place” policy.^15^ This means that older persons are supported to live independently at home as long as possible. To be effective, this policy should support informal caregiving and facilitate availability of sufficient formal care resources for care dependent older persons. Moreover, such policy is likely to be most effective if resources are allocated in a fair and equal way providing most to those with highest and complex needs.

We are not aware of previous studies that explore (in)equality of home care provision based on individual patient data and cover multiple European countries.

The main objective in this study was to compare the provision of home health care for older care dependent persons and explore signs of (in)equality of home health care allocation across organizations and across countries.

## Methods

### Design and setting

This study is part of the cross-European “Identifying best practices for care dependent elderly by benchmarking costs and outcomes of community care” (IBenC) project that was funded by the 7th Framework Program of the European Commission. The design of the IBenC study is described in detail elsewhere (www.ibenc.eu). In short, IBenC was conducted in 6 countries (Belgium, Germany, Finland, Iceland, Italy, and the Netherlands) and used a prospective longitudinal design, with assessments of care recipients. We used the baseline data only. In addition, country and local health care characteristics were collected by country reviews, meta-data, and expert advice.^1,16^

The study was approved by authorized medical ethical committees in the participating countries, and, where required, participants provided (written) informed consent before inclusion.

### Sample

#### Home health care organizations

The IBenC study focused on care recipients of home health care, defined as “care provided at home by social and/or health care professionals.” A home health care organization was defined as a professional care organization that offers nursing care (activities of nurses that are of technical, supportive, or rehabilitative nature), personal care (assistance with activities of daily living [ADL], such as dressing upper and lower body, eating, personal hygiene, toilet use, and bed mobility), and/or domestic care (help with instrumental activities of daily living [IADL], such as shopping, meal preparation, ordinary housework, transportation, managing medications, and managing money) in the community (www.ibenc.eu).

We aimed for a diverse sample of care organizations. Principal investigators from the 6 countries purposely recruited organizations in both urban and rural areas, large and small organizations, profit and not-for profit, independent teams versus embedded in primary care teams. For some countries, the study samples are, therefore, not necessarily representative of the total home care population in that country. Germany and Belgium have a relatively high number of small agencies and therefore included a higher number of agencies. In Iceland, the Netherlands and Italy bigger organizations predominate that cover large areas and whole cities. They included fewer agencies.

*Care recipients* were 2884 community-dwelling adults receiving care from the participating organizations, aged 65 years or older, expected to remain care recipient of a home health care organization at least 6 months after inclusion. We excluded 162 persons whose records indicated that they had not received any formal care in the 7 days preceding the assessment, and persons with missing items that were necessary to calculate their case mix index (CMI). This left us 2718 persons for the analyses (94.3%).

### Procedure

Care recipients from participating care organizations who fulfilled the inclusion criteria were invited in writing to participate. Italian participants and participants from 1 Dutch site were enrolled as anonymized routine care recipients and in accordance with local ethical regulations. Participants from these sites therefore reflected “real” caseloads, without potential selection because of study refusal. Client assessments were conducted in the homes of the clients by trained (research) nurses.

### Measurement and outcomes

#### Care recipients

Data on care recipients and resource use were collected by trained personnel with the interRAI Home Care (HC version 9.1.2), a comprehensive and structured assessment of medical, psychosocial, functional capabilities, and service use.^17^ The assessments included observations, information from medical records, and interviews with clients and informal caregivers. The interRAI-HC is part of a suite of instruments; all items from the interRAI suite have previously been shown to produce good inter-rater reliability across countries and setting.^18^ Content, construct, convergent, and predictive validity of the main functional scales and risk indicators that are embedded in this instrument were extensively tested in multiple national and multinational studies and reviewed by Poss et al.^19^ Validated language versions of the interRAI-HC were available in all languages required (www.interRAI.org).

*Functional status* was evaluated with the Activities of Daily Living Hierarchy (ADLH) scale, ranging from 0 (no impairment) to 6 (total dependence).^20^ The scale groups ADLs according to stages in the disability process. A score of ⩾3 indicates that extensive ADL support is required.

*Cognitive status* was assessed with 7-point Cognitive Performance Scale.^21^ A cutoff score of ⩾3 was used to indicate the presence of moderate to severe impairment.

*Care recipients’ characteristics* included gender, age, living alone, presence of informal carers, functional limitations, cognitive functioning, and mood.

*Formal care time* was expressed in total hours of professional care provided over 7 days preceding the assessment. This comprised 3 home care categories: home health aide, personal care, and nursing care, as well as 4 specialized therapies comprising physical, occupational, psychological, and speech therapy.

*Informal care time* included the total number of hours of informal care and active monitoring over the 3 days preceding the assessment provided by all family, friends, and neighbors. We extrapolated this amount to 7 days to match the time frame with provided formal care time. In the Belgian sample, this item was omitted in their interRAI-HC assessment. We therefore used the informal care estimate from the Resource Utilisation in Dementia (RUD) Lite measurement which was available in a Belgian subsample only (n = 83).^22^ The time frame for measuring informal care hours in the RUD Lite was 3 months preceding the assessment which we divided by 13 to have the same time frame of 7 days for the whole sample. Van Lier demonstrated satisfactory convergent validity between informal care estimates of interRAI-HC and RUD Lite assessments previously.^23^

#### Case mix index

To be able to make a fair comparison of resource use by patients across organizations, we applied case mix weights based on interRAI’s Resources Utilization Groups (RUG-III-HC; Appendix 1).^24^ The CMI is a relative weight that indicates the amount of formal care resources needed to meet a home care recipient’s needs in comparison with the average amount of care across all care recipients.^24,25^ Case mix index weights were derived from a large Ontarian sample of home care recipients that considered both volumes of formal and informal care.^19,26^ The RUG-III-HC distinguishes 7 main and 23 utilization subgroups based on patients’ clinical and functional characteristics that previously explained between 37.3% and 43.5% of the variation in resource use of formal and informal care in various countries.^24,27^ We used the latest Ontario CMIs that considered both formal and informal care (CMIFI) that were updated in 2014.^26^ These CMIs ranged between 0.49 for persons in the lowest RUG-III-HC intensity group, needing physical assistance only, up to 5.15 for persons in need of multiple extensive services (Appendix 2).^24,26^

#### Equality indicators of formal care hours

1. *Expected formal care time* was calculated by multiplying an individual recipient’s CMI with the average formal care hours of all recipients that corresponded to a CMI of one. The latter was calculated by dividing the average of formal hours (5.32) by the avarage CMI (1.316) of the whole group, (which is 4.04 hours). This approach was earlier described by James et al.^28^
2. *Difference of provided and expected formal care hours*: This metric expresses the extent to which provided and expected formal hours diverge or converge. Values close to 0 indicate relative convergence of provided and expected formal care hours. Negative values, or values below the country’s mean, indicate fewer than expected hours. Positive values, or values above the country’s mean, indicate more than the expected hours.

### Analyses

We *described* the sociodemographic and clinical characteristics of individual care recipients. For the remainder, aggregated data of care recipients of organizations and countries were the principal unit of our analyses. We calculated and plotted organizations’ and countries’ care recipients CMI and distribution over the 7 main resource utilization groups of care need (RUG-III-HC). Next, we calculated and plotted the distribution of formal care time differentiated by disciplines or type of care (home health aide, personal care, and nursing care, and physical, occupational, psychological, speech therapy).

*Equality* in formal home health care provision was explored by comparing and benchmarking differences between provided and expected formal care time across organizations and countries and plotting the differences including 95% confidence limits (computed by + and −1.96 times the SE). Signs of inequality *within countries* were considered present if confidence intervals or organizations did not overlap with their country’s confidence interval. Similarly, signs of inequality *between countries* were considered present if confidence intervals of countries did not overlap. To understand whether informal care played a role in the number of formal hours, we calculated associations between formal, informal, and expected formal care time using Spearman rank tests because of the skewed distributions.

SPSS statistical software, version 21, and Microsoft excel were used to calculate and visualize the results.

## Results

### Health care context of countries in the IBenC study

Table 1 describes characteristics of the 6 participating countries, including their coverage by health professionals, number of persons in long-term care at home, and typology of home health care. Italy and Germany had relatively aged populations, while Iceland had the youngest expressed by old age dependency ratio. All 6 countries are economically relatively well-off as shown by their purchasing power scoring at or above the European average. The share of persons with long-term care at home varied between 0.6% in Germany and 4.8% in the Netherlands.

**Table 1.** Overview of country, health, and social care context of participating countries.

	Belgium	Finland	Germany	Iceland	Italy	Netherlands
Population^a^ (million)	11 .1	5.4	80.5	0.32	59.6	16.7
Persons per km²^a^	364.3	17.7	229	3.2	201.5	494.5
Purchasing power^a^	119	115	122	113	99	129
Old-age dependency ratio: persons ⩾65 divided by 18-64 years^a^	26.4	27.7	31.2	18.9	31.6	24.4
Government health expenditures (% GDP)^b^	10.8	9.5	11.2	8.6	9.0	8.5
GPs/physicians/nurses per 1000 inhabitants^c^	1.11/2.9/15.4	NA/3.7/10	0.66/3.7/11	0.58/3.5/8	0.76/4.1/5.5	0.73/3.0/11.8
Persons with long-term care at home, N (%)^d^	152 318 (1.4%)	70 529^a^ (1.3%)	504 232 (0.6%)	NA	476 223 (1%)	610 180 (4.8%)
Eligibility for nursing care^e^	Doctor referral, nurse assesses ADL dependency	By municipality: criteria vary, mostly nurse assessment	Doctor referral: (I)ADL dependency ⩾6 months and LTC insurance	Doctor or municipality referral and nurse assessment	Doctor referral, needs tested, and availability informal care	Doctor referral, ADL dependency, general functioning, home environment
Personal care	By providers: capacity tested and availability of informal care	By municipality: criteria vary, mostly nurse assessment	(I)ADL dependency ⩾6 months and LTC insurance	By municipality	GP’s certificate: Means and needs tested	By national criteria, including availability of informal care
*National surveys: Imagine an older father or mother living alone who can no longer manage to live without regular help because of his or her physical or mental health condition. What would be the best option?¶*						
Live with their children, % (n)	17 (1040)	7 (1026)	25 (1510)	Na	28 (1017)	4 (1001)
Public authorities should provide appropriate care for elderly people in need (%)	97	99	91	Na	88	96
Typology of home health care (ANCIEN 2012)^1^						
Informal care oriented, low private financing	X		X			
Informal care oriented, high private financing		X				
Generous accessible and formalized				X		X
High private financing, informal care necessity					X	

Abbreviations: ADL, activities of daily living; GDP, gross domestic product; IADL, instrumental activities of daily living; GP, general practitioner; LTC, long term care; ANCIEN, assessing needs of care in european nations study; NA, not available.

GDP is an indicator of economic activity defined as a value of all produced goods and services without the value of any goods or services used in their production. Purchasing Power Standards (PPS) is expressed in relation to the European Union (EU28) average set to equal 100.

a Inhabitants, surface, and age distributions, PPS on January 1, 2013 (Eurostat): https://ec.europa.eu/eurostat.

b Government Expenditure on Health in 2011, expressed in % of GDP (Eurostat 2011).

c Organisation Economic Co-operation and Development Health data 2013, based on data from 2011 (Dutch nurse data from 2010). Finnish data were derived from the Finnish Medical Association (http://www.laakariliitto.fi/tutkimus/laakarityovoima/) and Finnish Nurses Association (http://www.sairaanhoitajaliitto.fi/viestinta/tilastoja/), both 2013. In Belgium, Germany, Iceland, and Italy, the data refer to practicing physicians and nurses providing care directly to patients. In the Netherlands and for nurses in Italy, the data refer to professionally active physicians and nurses, including 5% to 10% working as managers, educators, and researchers.

d This includes the “regular clients” with long-term care in Finland. In the official statistics, only those (1) with a care plan and (2) with a visit minimum once a week are calculated as regular clients.

e This indicator was part of the European Health Care Index 2013 and graded on a 3-level scale—good, so-so, not-so-good—based on the percentage (%) public spending of the total health care.

More Germans (25%) and Italians (28%) find that children should take care of their dependent parent compared with Finnish (7%) and Dutch (4%) people. Almost all find that public authorities should offer appropriate services for older persons in need.

Although eligibility criteria vary for home nursing and personal care, a certain level of need and functional dependency was required in all countries and municipalities. In most countries, nurses perform eligibility and allocation assessments. Belgium, Italy, and the Netherlands take availability of informal care into account. Recipients from Italy are also means tested for personal care.

### Organizations

In total, 33 home care organizations were included. Most organizations provided both nursing and social care. All Belgian and 2 Dutch organizations mainly provided nursing care. The organizations in Belgium, Germany, and the Netherlands were private organizations, mostly not for profit. The organizations in Italy, Iceland, and Finland were owned by the government or by a municipality. Six of the German organizations and all 3 Dutch organizations were administratively independent. The other organizations were embedded in a larger care organization.

### Home health care recipients

A total of 2718 eligible home care recipients served by 33 home care organizations were analyzed for this study. We had relatively equal sample sizes per country: 525 (18.2%) from Belgium, 456 (15.8%) from Finland, 493 (17.1%) from Germany, 420 (14.6%) from Iceland, 499 (17.3%) from Italy, and 491 (17.0%) from the Netherlands.

Two-thirds of the home health care recipients were female; the average age was 82.9 (SD = 7.3). The majority of the participants lived alone (57.8%). Significant country differences were seen between the country-specific samples: moderate to severe cognitive impairment (Cognitive Performance Scale (CPS) > 2) ranged from 1.9% in the Netherlands up to 37.1% in Italy. Regarding impairment in activities of daily living (ADL), 56.0% scored 1 or higher on the ADL hierarchy scale. This ranged from 16.8% in the Netherlands to 96.3% in Belgium.

### Distribution of the main resources utilization groups across organizations

**Figure 1.**
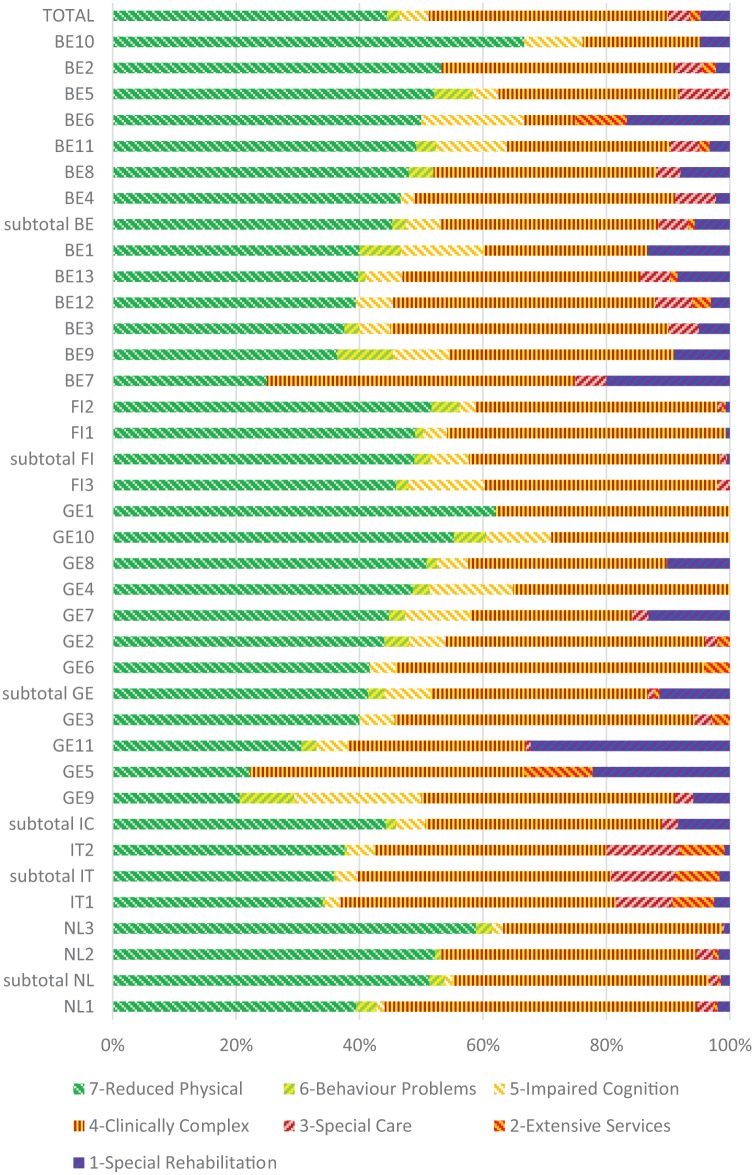
Distribution within organizations and countries of the 7 main categories of the interRAI Resource Utilization Groups (RUG-III-HC). Resource intensity is sorted on the proportions within countries on the least resource intensive category “reduced physical” (7: dark green).

Figure 1 illustrates the distribution of care recipients over the 7 main branches of the resources utilization groups. Most care recipients were classified in the reduced physical function category (RUG groups PA1 to PC0 in Appendix 1) (44%) indicating relatively low resource intensity with assistance in ADL and IADL tasks. Next best populated category concerned clinically complex care (RUG groups CA1 to CC0 in Appendix 1) (38%). This concerns more resource intensive nursing care such as for dehydration, chemotherapy, blood transfusion, urinary tract infection, dialysis, or end-stage disease. In 3 organizations, a relatively large portion of care recipients were classified in the rehabilitation category (RUG groups RA1 to RB0 in Appendix 1). This category is populated with recipients who use at least 2 h of occupational or physiotherapy during the last week (GE5,GE11; BE7).

### Distributions of informal and formal care provision across organizations

**Figure 2.**
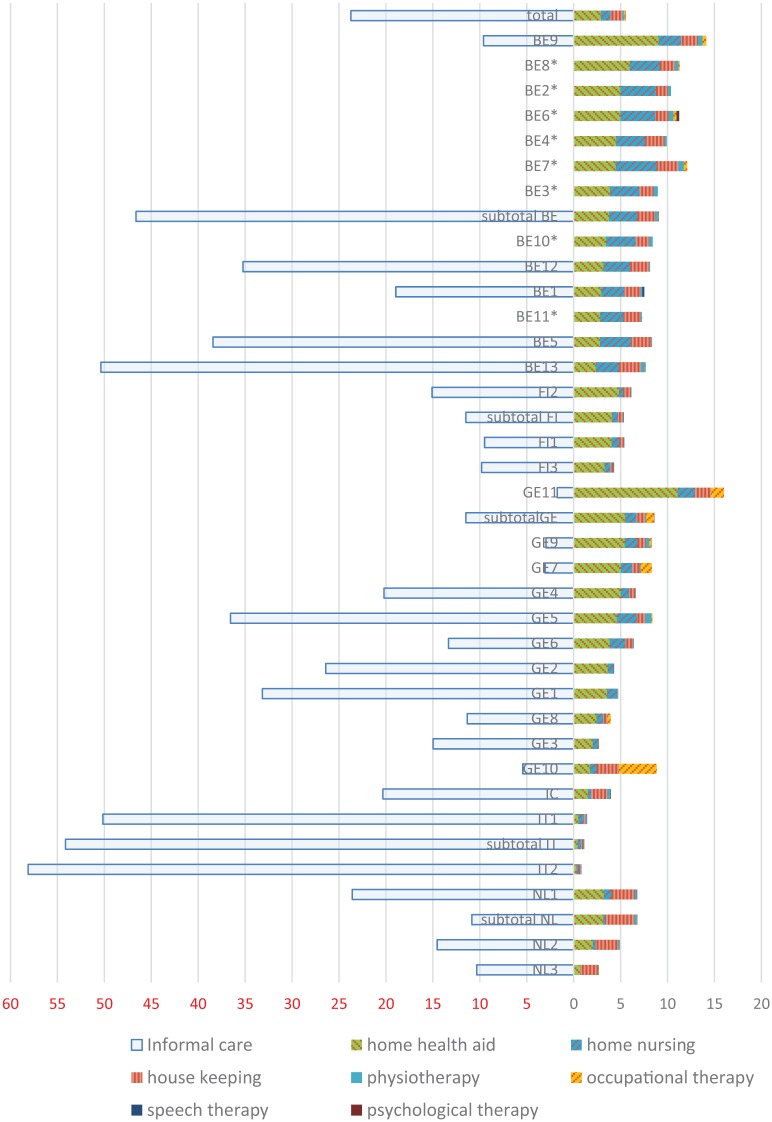
Distributions of informal and formal care hours in the last week by community care professionals across organizations, sorted within countries on home health aid (green).

Figure 2 shows the informal and formal care hours over the last 7 days across the organizations and countries.

The average number of formal care hours including the paramedical disciplines was 5.32 (SD = 5.91). This ranged from on average 1.02 h for Italian recipients to 9.01 h in Belgium. The average of formal care hours without paramedical disciplines (so only nursing care, home health aide, and domestic care) was 5.05 (SD = 5.65) hours per week. For the 2 Italian organizations, the expected and provided formal hours rather diverge.

For 2370 persons, presence of informal care was recorded and this was on average 23.48 h (SD = 37.60), Italian home care recipients received most informal care, 53.26 h (SD = 39.24), and German recipients the least, 11.71 h (SD = 27.12)

Nursing care, home health aide, and domestic care covered 95% of all formal hours (range from 84.1% for “Germany 7” to 99.4% for “Finland 2”). Over half of all formal hours concerned support by home health aides who typically provide ADL and some IADL assistance (53.6%). Support for housekeeping concerned 23.6%, and home nursing such as wound care or technical nursing support accounted for 17.9% of the formal hours.

Paramedical care accounted for 5% of the formal hours. Physiotherapy was the largest paramedical provider accounting for 3% of all formal hours.

### Provided and expected formal and informal care

**Figure 3.**
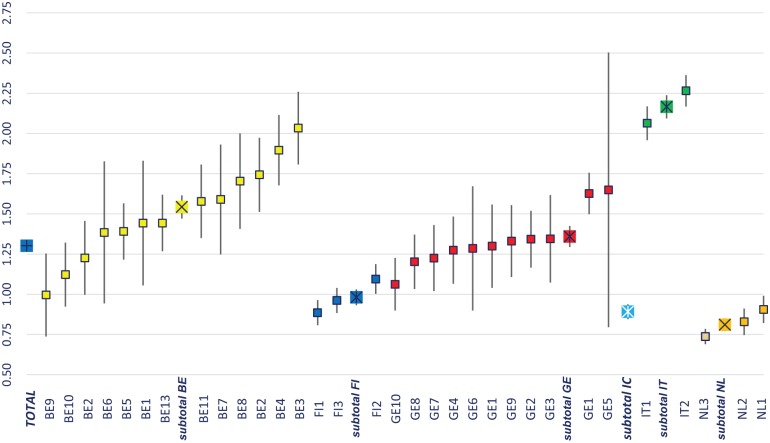
Benchmarking case mix index across recipients from 33 agencies and 6 countries. Sorted from low to high CMI within countries. Lines represent 95% confidence intervals.

Figure 3 illustrates the average recipients’ case mixes on organization and country levels. The average CMI was 1.316 (SD = 0.82) and varied between 0.90 in the Dutch sample up to 2.16 in the Italian sample. This indicates that Italian recipients on average were much more resource intensive compared with recipients from the other countries.

Provided formal care time was modestly and inversely associated with informal care time (Spearman’s rho = –0.29, *P* < .0001). This association was present in Belgium, Germany, and the Netherlands, but almost absent in Finland, Iceland, and Italy. Care recipients’ expected formal care time was associated moderately strong with informal care time (Spearman’s rho = 0.42, *P* < .0001). This association was absent in Germany and very small in Finland. Expected hours were strongly associated with formal and informal care hours summed up (Spearman’s rho = 0.58, *P* < 0001), except for Italy where this association was absent.

### Equality of formal care provision across organizations and countries

**Figure 4.**
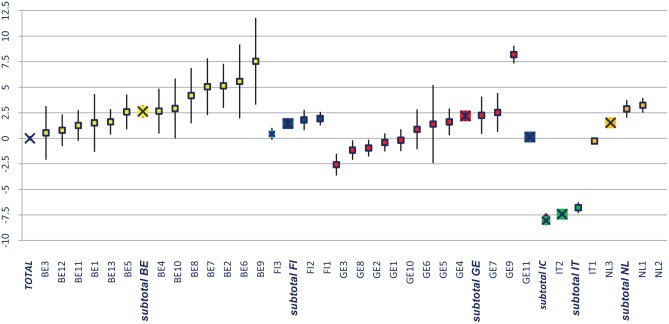
Benchmarking the difference between provided and expected formal care hours for care recipients of 33 organizations from 6 countries with 95% confidence intervals. Sorted on small to large across organizations within countries. The zero line represents the whole sample’s benchmark value of 5.32 formal hours.

Figure 4 benchmarks the difference between the provided and expected formal care hours for recipients from organizations in 6 countries (colored). The *Y*-axis represents the differences between the expected and provided formal care time. The zero line represents the whole sample’s benchmark value of 5.32 formal hours.

Substantial between- and within-country differences were found. Most Italian care recipients received (much) less formal care compared with the expected amount.

We also found substantial variations within countries with organizations providing more or less hours than expected. For example, 2 Belgian organizations fell out of the 95% confidence limits and provided more hours than expected in the Belgian sample. In Germany, 5 of 11 organizations fell outside of their country’s sample average, 4 provided less, and 1 provided far more than the expected hours. In both the Netherlands and Finland, 1 organization provided significantly less than their country’s average. The differences between the 2 Italian organizations remained within the confidence limits of their country’s average.

## Discussion

The main objective of this article was to explore signs of (in)equality in the provision of home health care for older community care recipients from multiple organizations in 6 European countries. We benchmarked expected and provided formal care time of older home health care recipients of 33 care organizations across 6 countries. Expected care time was estimated by multiplying individual recipients’ CMI with the average hours of provided care of the whole sample.

We found substantial differences between provided and expected formal care time both within and across countries. This implied that the equality of allocation of home care provision may be challenged.

In 4 out of 6 countries, significant and substantial differences were found between the hours of provided and expected care. Such differences were relatively modest within the Finnish and Italian samples, but clearly present across the Dutch organizations, and rather substantial across German and Belgian organizations.

The number of informal care hours was associated stronger with care recipients’ expected formal hours than the provided formal care hours. This may indicate that informal carers respond more quickly to care recipients’ care needs. Informal care exceeded formal care by far in all organizations and countries.

Remarkably, care recipients from both Italian organizations received relatively little formal care At the same time, they had the heaviest case mix with relatively many recipients with clinically complex needs, and persons needing extensive services. This may partly explain the high amount of informal care for the Italian care recipients. It does question how adequate these more complex care recipients were served and whether their informal carers experienced more burden.

The *variations within some countries* indicate that their allocation procedure may produce inequalities in home health care provisioning. Comparable observations were made previously provision.^29^ It questions whether allocation procedures allow such room for professionals or organizations to apply criteria in a more generous or strict way.

Several alternative explanations are possible. In Germany, Italy, and Finland, allocation of home care is the responsibility of the federal states, regions, and municipalities, respectively, and their allocation procedures vary. Another explanation may be related to financial reimbursement. For example, reimbursements for home health care in the Netherlands allow a maximum of 16 h a week, above which a person should apply for the long-term institutional care. If a person refuses institutionalization, home health care organizations may temporarily provide more hours than they get reimbursed. Next, relatively wealthy care recipients are able to purchase additional services out of pocket easier and therefore consume relatively more formal time. Also, German organizations can apply for additional funding for occupational therapy, but frequently do not because of the bureaucratic hassle.^30^

*Variations across countries* were much larger, especially between Italy and the other countries. It is not easy to label this as inequality. Variations can be expected across countries or health jurisdictions as allocation criteria differ as well as the availability of formal or private resources. Also, cultural expectations and legal requirements about involvement of informal care may produce a different balance between formal and informal care.^2^ In addition, for example, in Italy, families frequently contract external foreign workers to support their relative. Their care time was registered under informal care, which explains the very different balance between the amount of formal and informal care. Informal caregivers may provide adequate support for assistance with IADLs and perhaps also several ADLs. Nevertheless, to care for the clinically complex needs, involvement of a skilled health professional seems more appropriate.^31^ We did not look at possible (negative) consequences for informal carers. There is a large body of literature on substantial informal care burden especially in caregivers of recipients with dementia.^7,10,11,32^ Wagner and Brandt^33^ reported on better spousal caregiver well-being in European regions with more formal care resources.

The (modest) negative association between informal and formal care supports earlier observations by Bolin et al. They reported that informal and formal home care are substitutes, although these relationships may differ according to the complexity of the care needs, as well as a European north-south gradient.^9,34^

A *strength* of this study was that care recipients from 6 Western European countries were included with sufficiently large samples to capture meaningful variations. Next, the quality of the data was high as trained assessors used an internationally validated and reliable measurement, the interRAI-HC.^18^ In addition, we used a sophisticated and well-validated case mix measure, which allowed us to make valid comparisons across organizations of expected formal care hours.

A first *limitation* is that is we remain uncertain about the *representativeness* of our samples. We included only a limited number of organizations per country and selected on diversity of their location, size, care type, management, or payment form. Sites who used research sampling likely experienced selective sampling. For example, 2 sites in the Netherlands recruited only a limited number of persons with cognitive impairment because it was difficult to seek informed consent from the legal representative. In Germany, people living in assisted living and residences were overrepresented compared with their national average of 10%.^30,35^

On the other hand, the dependency levels in our samples closely reflect previously reported dependency levels on home health care recipients in several European countries, including Germany, Iceland, the Netherlands, Italy, and Finland.^13^ Also, comparison of the Icelandic and German study samples’ dependency levels did not differ from their national reference data.^30,35,36^ In addition, the 2 Italian and 1 Dutch samples were drawn from routine practice, and therefore reflect care recipients from those organizations very closely. From several countries, we included only few (bigger) organizations and even one from Iceland. We did not look at differences within organizations. With more organizations, perhaps even more differences would have been found in expected and provided care.

A next limitation is that we remain uncertain about the validity of the Ontario case mix weights for our diverse European care contexts. The distribution of participants across the main resource utilization groups was fairly comparable with the routine care sample of Ontario as well as a previous research sample from Michigan.^24,25^ Also, it is unlikely that ADL support or treatments by nurses from these 2 North American countries would take much more or much less time than professionals from European countries would need. Nevertheless, we did see substantial differences between average observed care time across countries.

The rather substantial differences of provided professional care time lead to the question of how nurses across countries manage their time, especially when they lack time. Do Italian care professionals delegate tasks to informal carers or au pairs? Do recipients with clinical complexities in low resourced contexts more often end up in hospitals?

A last limitation is that we did not distinguish between type of expected formal care. A differentiation of expected hours across nursing, health assistance, and domestic and paramedical care would have been perhaps more informative.

*Future study* may validate and calibrate the case mix weights in our diverse European care contexts to improve the estimates for expected care. Next, we need to better understand how informal and formal home health care interact, and what an optimal balance would be for both care recipients and informal carers. This also touches on a more fundamental issue whether (in)equality and (un)fair allocation affect patient outcomes, informal carers’ well-being, and costs of care. These issues will be addressed in future IBenC publications. Finally, as the interRAI-HC is used in home care sites all over the world, replication and expansion of this study is possible at relatively low research cost. This may provide more robust estimates of the inequalities we observed.

## Conclusions

We found substantial variations in provided formal care time among organizations both within and across countries that could not be explained by the case mix differences of recipients. This implied the presence of inequality of allocation of home health care. These findings may alert professionals and policy making striving for equal home health care provisioning for dependent older persons.

## Figures and Tables

**Figure:**
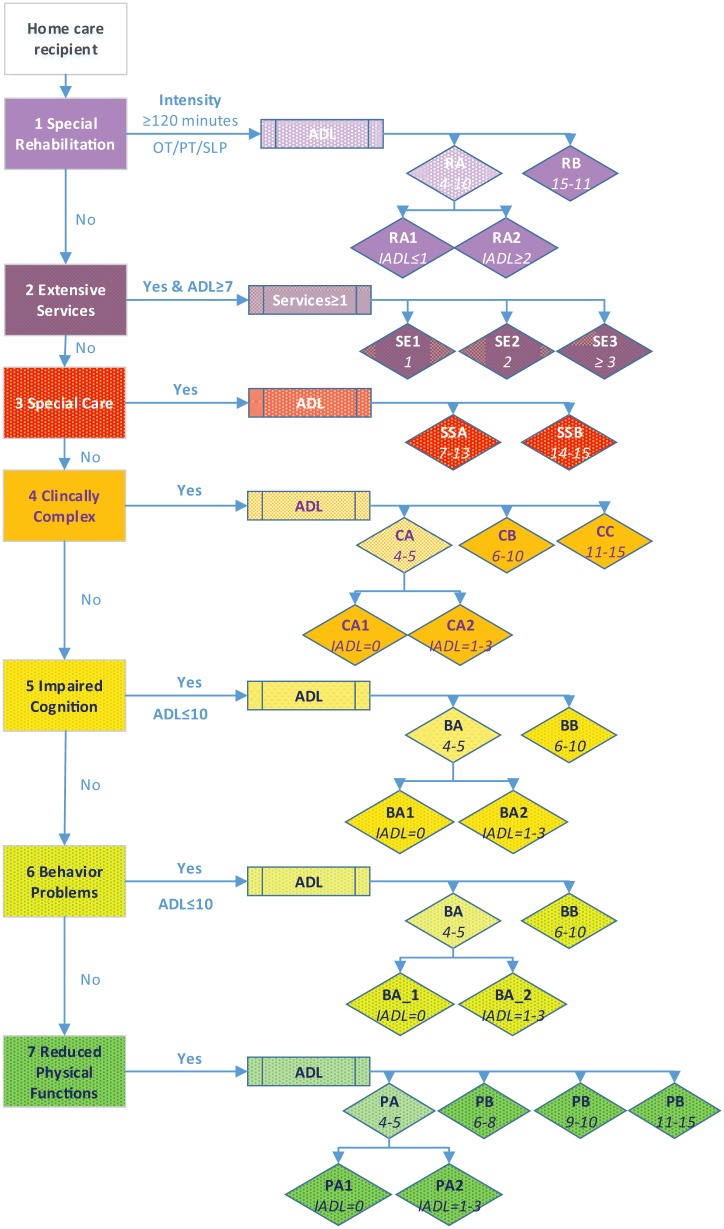
Algorithm Resources Utilization Groups of home care recipients (reproduced from Poss et al.^19^).

## Appendix 1

**Criteria**

**1 Special rehabilitation**
  ⩾120 min of physical-, occupational-, speech/audiology-, or psychological therapy in the last 7 days
2 Extensive services
  Tracheostomy, respirator, or respiratory therapy
3 Special care
  Stage 3 or 4 pressure ulcer
  Enteral tube feeding
  Diagnosis of multiple sclerosis
  Treatment for burns
  Radiation treatment intravenous
Fever and one or more of
   -Dehydrated
   -Diagnosis of pneumonia
   -Vomiting
   -Unintended weight loss
4 Clinically complex
  Dehydrated
  Any stasis ulcer
  End-stage
  Chemotherapy
  Blood transfusion
  Skin problem
  Diagnosis of cerebral palsy
  Diagnosis of urinary tract infection
  Diagnosis of hemiplegia
  Dialysis treatment
  Diagnosis of pneumonia
  One or more of 7 criteria in special care
  One or more of 3 criteria in extensive services
5 Impaired cognition
  Cognitive Performance Scale ⩾3 (~ = Mini Mental State Examination ⩽ 15)
6 Behavior problems
  Wandering
  Verbally abusive
  Physically abusive
  Socially inappropriate
  Hallucinations
7 Reduced physical functions
  All others
ADL = Activities of daily living score ranges between 4 and 15 (eating, toilet use, transfer, bed mobility).
IADL = Instrumental activities of daily living score ranges between 0 and 3 (meal preparation, medication management, phone use).

## Appendix 2

### Supplementary data on case mix index

Case mix index (CMI) values were derived from a large routine sample of home care recipients from Ontario in which their RUG-III-HC needs profiles were linked to claims data and actual resource use. These CMIs reflect an update from the publication of Poss et al^19,26^ from 2008. As informal career was present in the majority of the IBenC sample, we applied the CMI weights that assume the presence of both formal and informal care (last column). Colors correspond with the RUG-III-HC figure in appendix 1.

**Table:**

RUG III HC groups	N	Formal CMI	Formal + informal CMI
1 Rehabilitation RB	1.724	3.03	2.74
RA2	4.508	1.53	1.61
RA1	8.671	1.16	0.97
2 Extensive services SE3	75	8.90	5.15
SE2	258	6.18	4.24
SE1	2.586	2.67	2.50
3 Special care SSB	2.436	2.97	2.79
SSA	4.118	2.12	1.88
4 Clinically complex CC	4.834	2.44	2.59
CB	11.969	1.66	1.66
CA2	43.816	1.13	1.13
CA1	40.652	0.89	0.61
5 Impaired cognition IB	7.456	1.53	2.12
IA2	12.694	1.11	1.64
IA1	316	0.90	0.84
6 Behavior problems BB	603	1.32	1.72
BA2	2.041	0.92	1.28
BA1	601	0.73	0.59
7 Reduced physical functions PD	11.482	2.13	2.42
PC	4.763	1.59	1.76
PB	15.228	1.22	1.38
PA2	110.631	0.83	0.93
PA1	114.790	0.61	0.49
Total	406.252	1.00	1.00
